# The Effect of Using Virtual Reality on School-Age Children’s and Caregivers’ Anxiety in the Emergency Room: True Experimental Study

**DOI:** 10.2196/74900

**Published:** 2025-08-26

**Authors:** Enjelita Karujan, Dessie Wanda, Efa Apriyanti

**Affiliations:** 1 Faculty of Nursing, Universitas Indonesia, Gedung Pendidikan dan Laboratorium, Jl. Prof. Dr. Bahder Djohan, Depok, 16424, Indonesia, 62 85695180195

**Keywords:** anxiety, caregivers, children, emergency room, virtual reality

## Abstract

**Background:**

Being treated in an emergency room (ER) could be a stressful experience and trigger anxiety in children. Virtual reality (VR) is a technology-based distraction technique that can be used for school-age children.

**Objective:**

In this study, we aimed to identify the effect of using VR in reducing anxiety in school-age children in the ER and identify the relationship between caregivers’ and school-age children’s anxiety.

**Methods:**

This study employed a true experimental design using a post-test-only control group involving 66 children aged 6‐12 years, randomly selected according to the inclusion and exclusion criteria. The intervention group, consisting of 33 children, received VR intervention, and the control group, consisting of 33 children, received standard care. Three respondents dropped out. Data analysis employed descriptive statistics, independent *t* tests, one-way ANOVA, and Pearson correlation analysis.

**Results:**

Most respondents were boys (39/66, 61.9%), accompanied by their mother (34/63, 54%), and had prior experience admitted to the ER (31/63, 49.2%). The anxiety in school-age children in the intervention group (mean 17.71, SD 3.013) was lower than that in the control group (mean 22.31, SD 3.167). There was a significant difference in the anxiety mean scores between the intervention group and the control group (*t*_(61)_=−5.907, *P*<.001). The mean (SD) of the caregivers’ anxiety in the intervention and control groups were 46.06 (9.413) and 54.44 (9.112), respectively. There was a moderate relationship between caregivers’ and school-age children’s anxiety *(r*=.532, *P*<.001).

**Conclusions:**

This study has proven that VR can reduce school-age children’s anxiety.

## Introduction

Being admitted to the emergency room could be a stressful experience and could cause anxiety in children and their families [1]. When entering the emergency room, children had unpleasant experiences due to the bright lights, unusual sounds, different smells, strangers, and busy environments [2]. A study showed that 77.4% of school-age children being treated in the emergency department experienced anxiety [3]. Nursing interventions, such as distraction techniques, are needed to reduce anxiety and improve comfort for children. Distraction is a non-pharmacological technique that can be used to reduce anxiety in children that is easy to implement and effective in reducing anxiety [4].

VR is one of the distraction techniques where children are given goggles and a headset to view a virtual display from a computer [5]. A study conducted on 142 children aged 7‐12 years who received venipuncture showed that VR was effective in reducing children’s anxiety [6]. This result is in line with the results of research conducted on 40 children aged 6‐12 years who will undergo abdominal surgery, which showed that VR was effective in reducing preoperative anxiety levels [7].

Watching a video using VR involves more than one sense, vision, and hearing, making VR more attractive to hold on children’s attention compared to other techniques that only involve one sense, such as music (hearing) or picture books (vision) [8]. The presence of parents can be a source of motivation and can strengthen children [9]; therefore, parents’ anxiety relates to children’s anxiety [10]. In light of this, a study about the use of VR to reduce anxiety in school-age children treated in emergency rooms is needed.

## Methods

### Study Design

This study was a true experiment study using a post-test-only control group design.

### Sample

This study involved 66 children aged 6‐12 years, with urgent and non-urgent triage, compos mentis (conscious), no respiratory or circulatory emergencies, no disturbance in the head and face area, and being able to communicate. The sample size was calculated using the sample size formula for hypothesis testing of the means of two independent populations based on literature [11]. The effect size was 3,4 based on previous research [12]. Samples were selected using simple random sampling by applying one drawing for each day of admission.

### Instrument

The instruments used were the demographic questionnaire, the short form of the Chinese version of the State Anxiety Scale for Children (CSAS-C) [13] to measure children’s anxiety, and the State Anxiety Inventory (SAI) [14] to measure caregivers’ anxiety.

### Intervention

After passing the triage assessment, respondents in the intervention group received a VR intervention for 10 minutes. They watched an animated video of Pororo the Little Penguin about the situation in the emergency room. The tool used was entry-level, low-immersive VR (smartphone and headset Sinecon VR Box).

### Data Collection

Data collection for this study was conducted between February until May 2023. After passing the triage assessment, the researcher assessed which respondents matched the inclusion and exclusion criteria. The researcher explained the purpose and benefits of the research as well as the procedures that will be passed by the prospective respondent and ask for their consent. The child’s anxiety was measured immediately after the intervention. Meanwhile, the companion’s anxiety was measured after the child completed the procedure in the emergency room.

### Data Analysis

The data were analysed using IBM SPSS Statistics 20, with a significance level of 0.05. Univariate analysis was performed using distribution of frequencies and percentages and mean-standard deviation for categorical and numerical data, respectively. The analysis of the children’s anxiety differences between the intervention and control groups used the independent *t* test.

### Ethical Considerations

Ethical approval was obtained from the hospital where the study was conducted (Ethical approval number: 017/EC/KEPK-KANDOU/II/2023). Consent was obtained from the accompanying persons and from the children, when possible. No names were provided by the respondents. Following completing the questionnaire, all respondents received a gift of US $2 from the researcher.

## Results

**Figure 1.**
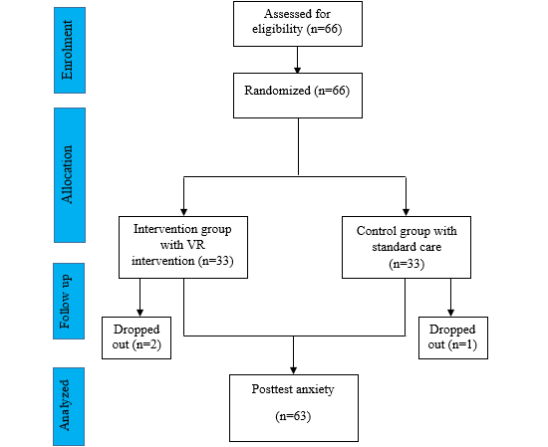
CONSORT diagram. VR: virtual reality.

Figure 1 describes the sampling technique applied. Sixty-six envelopes were provided, half containing a paper labeled “intervention” and the other half labeled “control”. Three respondents dropped out due to rejection on completing the questionnaire, making the total number of samples was 63. The participants were 63 school-aged children. Participant characteristics are presented in Table 1. Most of the respondents were boys (39/66, 61.9%), had been admitted to the emergency room in the hospital previously (31/63, 49.2%), and were accompanied by their mother (34/63, 54%).

**Table 1.** Distribution of respondent characteristics.

Variable	Intervention	Control	Total	*P* value
Sex, n (%)				.050^a^
Boy	17 (54.8)	22 (68.8)	39 (61.9)	
Girl	14 (45.2)	10 (31.2)	14 (38.1)	
Emergency care experience, n (%)				.614^a^
Never had experiences	5 (16.1)	6 (18.8)	11 (17.4)	
In the hospital where the research was conducted	13 (41.9)	18 (56.2)	31 (49.2)	
In another hospital, other than the research location	8 (25.8)	6 (18.8)	14 (22.3)	
In the hospital where the research was conducted and also in another hospital	5 (16.1)	2 (6.2)	7 (11.2)	
					Caregiver				.679^a^
Mother	19 (61.3)	15 (46.9)	34 (54)	
Father	1 (3.2)	1 (3.1)	2 (3.2)	
Mother and father	7 (22.6)	10 (31.2)	17 (27)	
Mother or father and other family	3 (9.7)	6 (18.8)	9 (14.3)	
Other family member	1 (3.2)	0 (0)	1 (1.6)	
Caregiver anxiety score, mean (SD)	46.06 (9.413)	54.44 (9.112)		.780^a^

a homogeneity test results with the Levene test.

The children’s anxiety in the intervention group (mean 17.71, SD 3.013) was lower than that of the children in the control group (mean 22.31, SD 3.167). There was a significant difference in the anxiety mean scores between the intervention group and the control group *(t*_(61)_=*−*5.907, *P*<.001). The effect size of this study was 4.6 (95% CI 6.161 to 3.045), which were higher than the effect size used as a reference, meaning that the use of VR was significant both from statistical and clinical reasons.

There was a moderate relationship between caregivers’anxiety and school-age children’s anxiety (*r*=.532, *P*<.001). The mean (SD) caregiver anxiety was 46.06 (9.413) in the intervention group and 54.44 (9.112) in the control group.

## Discussion

### Principal Findings

The use of VR could reduce anxiety in school-age children treated in the emergency room (*P*<.001). This result coincides with the study involving children undergoing surgery, which found a difference in preoperative anxiety between children who received VR intervention and those who did not (*P*<.05) [7].

VR is a diversion method where the child is given tools resembling glasses and headsets to view virtual images from a computer [5]. In the era of digital technology, VR can support nurses in implementing nursing care for patients [12]. These findings show significant differences in anxiety between the control and intervention groups. The mean score of the intervention group was lower than that of the control group. The children who received the VR intervention focused on the video they were watching [5]. Watching video using VR involves more than one sense, namely, vision and hearing. This made the use of VR more attractive to the children’s attention compared to distraction techniques that only involved one sense, such as music (hearing) or picture books (vision) [8]. The children enjoyed the video presented using VR; therefore, their anxiety was reduced.

There was a moderate relationship between caregivers’anxiety and school-age children’s anxiety (*r*=.532, *P*<.001). The presence of parents can be a source of motivation and strengthen children [9]. Parents’ anxiety is related to children’s anxiety [10]. When the child’s anxiety decreased, the parent’s anxiety also decreased.

### Limitations

This study did not measure the anxiety score before the intervention due to permission granted from the research location. The anxiety measurement before the intervention would delay the treatment that the patient would receive. Apart from that, the intervention was only given for 10 minutes, so the researchers were concerned that giving questionnaires twice, both for children and caregivers, in a short time interval could cause discomfort.

### Conclusions

The findings of this study show that the use of VR could reduce anxiety in school-age children treated in the emergency room. Therefore, the authors recommend that hospital management establish a policy related to the use of VR in managing children’s anxiety.

## Abbreviations

CSAS-C: Chinese version of the State Anxiety Scale for Children
SAI: State Anxiety Inventory
VR: virtual reality
